# Bone health following paediatric and adolescent bariatric surgery: a systematic review and meta-analysis

**DOI:** 10.1016/j.eclinm.2024.102462

**Published:** 2024-02-02

**Authors:** Anuja Tulip Mitra, Bibek Das, Khalid Maher Sarraf, Martha Ford-Adams, Matyas Fehervari, Hutan Ashrafian

**Affiliations:** a Department of Surgery and Cancer, Imperial College London, Hammersmith Hospital, Du Cane Road, London, United Kingdom; b Department of Orthopaedics, St Mary's Hospital, Imperial College Healthcare NHS Trust, Praed Street, Paddington, London, United Kingdom; c Department of Paediatric Endocrinology, Kings College Hospital NHS Foundation Trust, London, United Kingdom; d Department of Bariatric Surgery, Chelsea and Westminster Hospital, London, United Kingdom

**Keywords:** Paediatric, Adolescent, Bariatric, Bone, Calcium, BMD

## Abstract

**Background:**

Childhood obesity is a pressing health crisis of epidemic proportions. Bariatric surgery (BS) is an effective weight loss solution however its role in the paediatric population is contentious owing to the paucity of weight specific and generalised health outcomes. This systematic review and meta-analysis aimed to assess the impact of paediatric BS on bone health.

**Methods:**

This prospectively registered systematic review (PROSPERO ID: CRD42023432035) was performed in accordance with PRISMA guidelines. We searched MEDLINE (1946–1928 September 2023), EMBASE (1947–1928 September 2023) via the Ovid platform, and the Cochrane Review Library to identify scientific publications reporting bone outcome measures in patients under the age of 18 years who underwent BS. Meta-analysis was undertaken on post-operative weight and bone parameters in paediatric patients following BS. Outcomes were reported as weighted or standardized mean difference with 95 percent confidence intervals. Subgroup analysis by intervention, quality scoring and risk of bias were assessed.

**Findings:**

Twelve studies with 681 patients across 5 countries (mean age 17 ± 0.57 years) were included. The quality of included studies was rated as high and there was substantial between-study heterogeneity for most factors included in the meta-analysis (*I*^*2*^ from 0% to 99.1%). Patients underwent Roux-en-Y gastric bypass (RYGB, n = 216), sleeve gastrectomy (SG, n = 257), gastric band (n = 184) or intragastric balloon placement (n = 24). BS was associated with significant weight reduction, body mass index (BMI) −12.7 kg/m^2^ (95% CI −14.5 to −10.9, p < 0.001), with RYGB being most effective, BMI −16.58 kg/m^2^ (95% CI −19.6 to −13.6, p < 0.001). Patients who underwent SG or RYGB had significantly lower lumbar bone mineral density, −0.96 g/cm^2^ (95% CI −0.1 to −0.03, p < 0.001), Z score, −1.132 (95% CI −1.8 to −0.45, p < 0.001) and subtotal body bone mineral density, −0.7 g/cm^2^ (95% CI −1.2 to −0.2, p < 0.001) following surgery. This was accompanied with higher markers of bone resorption, C-terminal telopeptide of type 1 collagen 0.22 ng/ml (95% CI 0.12–0.32, p < 0.001) and osteocalcin, 10.83 ng/ml (95% CI 6.01–15.67, p < 0.001). There was a significant reduction in calcium levels following BS, −3.78 mg/dl (95% CI −6.1 to −1.5, p < 0.001) but no difference in 25-hydroxyvitamin D, phosphate, bone alkaline phosphatase, procollagen type 1 N propeptide or parathyroid hormone.

**Interpretation:**

BS effectively reduces weight in paediatric patients, but RYGB and SG may have adverse effects on bone health in the medium term. It is crucial to monitor and support bone health through appropriate nutritional supplementation and judicious follow-up. Long-term data is needed to fully understand the clinical implications of these findings on bone outcomes.

**Funding:**

10.13039/501100000265Medical Research Council (MRC), United Kingdom.

**Research in context**

**Evidence before this study**

We conducted a systematic search in MEDILINE, EMBASE and COCHRANE databases on all studies reporting calcium and/or bone mineral density (BMD) in paediatric patients (<18 years) undergoing any BS intervention up to September 28, 2023. We found 12 studies from 5 countries with a total of 681 patients with a mean age of 17 years. Studies were rated as good quality using the AMSTAR assessment with a low level of bias based on the Newcastle Ottowa scores. BS was associated with significant weight reduction, body mass index (BMI) −12.7 kg/m^2^ (95% CI −14.5 to −10.9, p < 0.001), with Roux-en-Y-gastric bypass (RYGB) being the most effective, BMI −16.58 kg/m^2^ (95% CI −19.6 to −13.6, p < 0.001). Patients who underwent sleeve gastrectomy (SG) or RYGB had significantly lower lumbar BMD, −0.96 g/cm^2^ (95% CI −0.1 to −0.03, p < 0.001), Z score, −1.132 (95% CI −1.8 to −0.45, p = 0.001) and subtotal body BMD, −0.7 (95% CI −1.2 to −0.2, p < 0.001) following surgery. This was accompanied with higher markers of bone resorption, CTX 0.22 ng/ml (95% CI 0.12–0.32, p < 0.001) and osteocalcin, 10.83 ng/ml (95% CI 6.01–15.67, p < 0.001).

**Added value of this study**

As far as we are aware this is the first systematic review and meta-analysis to report on the impact of paediatric BS on bone outcomes. Given the acute surge in paediatric obesity, BS has become a popular management strategy to this problem however it is imperative that clinicians, patients, healthcare commissioning bodies fully understand the risks and benefits of BS during the childhood years. Our study has identified the main demographics of patients undergoing BS during childhood demonstrating it is mainly undertaken during the adolescent years in Western countries with a slight preponderance in females. Furthermore, this study has demonstrated the most common types of BS interventions undertaken in this cohort of patients being malabsorptive procedures of sleeve gastrectomy and RYGB. Our study has also synthesised the biochemical and radiological parameters evaluating bone health following BS. Through our meta-analysis we have demonstrated that BS is highly effective at generating weight loss however there are adverse impacts on bone health outcomes putting these patients at higher risk of developing oestomalacia and a greater fracture risk. We highlight the need for guidelines shaping future practice that incorporates judicious follow up and post-operative nutritional supplementation with dietetic support in this group of patients undergoing BS to mitigate the long-term adverse impacts on bone health.

**Implications of all the available evidence**

Our study highlights the need for bone health and paediatric obesity to be reviewed as a matter of urgency. We have generated evidence on the safety and impact of paediatric BS, demonstrating adverse effects on bone health in this population. The implications from this study may have far reaching impacts on patients, clinicians, policy makers and wider society. Absorbing the results of this research may have practice changing impacts such as implementing a structured post-operative nutritional supplementation program for all children undergoing BS and highlighting the importance of careful selection of individuals who are suitable to undergo BS during childhood as they should demonstrate the ability and commitment to adhering to long-term nutritional supplementation. This study may also help to shape the pre-operative counselling of patients so they can be aware of these risks and alternative weight loss strategies such as monitored lifestyle programmes or medical interventions can be offered. Future research is essential in this field owing to its novelty, especially the necessity of long-term data beyond 5 years that is critical to evaluate if these intermediate risks translate to clinically significant adverse bone outcomes.

## Introduction

Childhood obesity is a health crisis of epidemic global proportions. Its growth has superseded adult obesity in the Western world and is predicted to rise to an estimated 254 million cases by 2030.^1^^,^^2^

The consequences of childhood obesity are disastrous, adversely affecting physical and mental wellbeing, reducing quality of life and shortening overall life expectancy.^3^ It is closely linked to the development and exacerbation of a plethora of obesity associated medical comorbidities, such as type 2 diabetes, cardiometabolic and respiratory dysfunction, and many others.^4^ Reports suggest those undergoing bariatric surgery (BS) as an adult, present with greater and more severe obesity related comorbidities if they suffered from childhood obesity, demonstrating the cumulative detrimental effects of this problem.^5^

In adults, BS is highly effective at achieving significant long-term weight loss and the amelioration of obesity associated medical comorbidities. BS interventions such as laparoscopic gastric band (LAGB), sleeve gastrectomy (SG) and Roux en-Y gastric bypass (RYGB) reduce, restrict and prevent the absorption of calories and vitamin intake generating a net energy deficit to illicit long-term significant and sustainable weight loss.^6^ Given that early weight loss demonstrates superior long-term health outcomes, there have been considerable efforts that have focussed on the treatment of childhood obesity. The mainstay of these interventions have been non-surgical, such as lifestyle modifications alone or in combination with medical therapies like semaglutide.^7^ Whilst these have demonstrated various degrees of efficacy, the only intervention with evidence of significant and sustained weight loss has been BS.^8^^,^^9^

International guidelines state that BS is safe in individuals under 18 years.^10^ In this population, BS most commonly occurs during adolescence although it has been performed in children as young as 5 years, demonstrating durable weight loss and effective resolution of comorbidities.^11^ In comparison to adults, paediatric BS has been reported to generate superior weight loss (∼25%) over conventional weight management strategies (∼2%) and is more effective at achieving diabetes and hypertension remission.^9^, ^10^, ^11^ Despite these benefits, the uptake has been underwhelming. This is unsurprising given the natural caution exercised when considering operative interventions in children compounded by the lack of high-quality long term data.^12^^,^^13^ Malabsorptive BS procedures are known to cause mineral and micronutrient deficiencies in adults, forming the rationale for these patients to have life-long vitamin and mineral supplementation.^14^ Given the adolescent years are a critical time for the accrual of trabecular bone mass and overall skeletal health, when 25% of peak bone mass is accrued,^15^^,^^16^ any disruption in vitamin homeostasis or nutrient balance may negatively affect bone health. Studies in adults have demonstrated harmful effects on bone health with both diet and BS induced weight loss,^17^^,^^18^ however these outcomes in the paediatric population are unknown. The aim of this systematic review and meta-analysis was to therefore determine the effects of paediatric BS on bone outcomes.

## Methods

### Search strategy and selection of studies

The review was prospectively registered on PROSPERO (CRD42023432035). A comprehensive literature search was performed in accordance with the recommendations of the preferred reporting items for systematic reviews and meta-analysis guidelines (PRISMA) to identify scientific publications reporting bone outcome measures in patients under the age of 18 years who underwent BS. Searched databases included MEDLINE (1946–1928 September 2023), EMBASE (1947–1928 September 2023) via the Ovid platform and the Cochrane Review Library. Reference lists of eligible articles were also hand-searched for additional publications. The full search strategy can be found in Supplementary File 1. All variations in the spelling, including truncated search terms using wild card characters and the “related articles” function were used in combination with the Boolean operators AND OR. The entire search was conducted by two independent reviewers (A.T.M. and B.D.) and a third reviewer (M.F.) was consulted when disagreements in study inclusion arose. Ethical board approval and informed consent from participants was not required for this systematic review and meta-analysis.

### Eligibility criteria

Titles and abstracts were scanned with full text review of relevant articles to evaluate for study inclusion. The inclusion criteria required articles to report on paediatric patients (aged between 5 and 19 years old as defined by American Society of Metabolic and Bariatric Surgery and International Federation for the Surgery of Obesity and Metabolic Disorders guidelines) with obesity defined as having a BMI >35 kg/m^2^ with a concurrent medical co-morbidity or BMI >40 kg/m^2^ alone, who underwent bariatric surgery procedures (any type of weight loss surgery). Data extracted were pre- and post- BS bone outcomes (calcium and/or bone mineral density).^10^ Comparative cohort studies, non-randomized prospective and retrospective studies, and randomised controlled trials were included. Case series, case reports, narrative reviews, editorials, and conference abstracts; or studies with fewer than five participants and publications in a non-English language were excluded.

### Data extraction and quality assessment

Information was extracted in duplicate and populated on a pre-specified data template. Outcomes included year of publication, study design, sample size, country of study, baseline demographics of patients, type of bariatric intervention, pre- and post-operative anthropometric data and the following bone parameters; calcium, phosphate, bone alkaline phosphatase, 25-hydroxyvitamin D (25OHD), parathyroid hormone (PTH), trace minerals, magnesium and zinc, osteocalcin, procollagen type 1 N propeptide (P1NP), C-terminal telopeptide of type 1 collagen (CTX), subtotal body and lumbar bone mineral density (BMD), lumbar bone mineral content (BMC) and fracture rate. All studies were appraised for quality and rigorousness using the Newcastle-Ottawa Scale (NOS) and JADDAD score^19^^,^^20^ and the methodological quality of the article was evaluated using the AMSTAR checklist^21^ (Supplementary File 2).

### Outcome measures

All data was extracted up to 24 months following BS. Data was pooled to for effect estimates for meta-analysis if there were 3 or more studies. Baseline characteristics are reported as mean values with standard deviations and percentages. Modulation between pre- and post-surgery groups are reported as standardized or weighted mean differences, with 95% confidence intervals.

### Statistical methods

All p-values are two-tailed with p < 0.05 considered statistically significant. The random-effects model was chosen for weight loss outcomes to account for data extracted from heterogenous populations and confounders that cannot be standardized on data pooling. In all cases, statistical heterogeneity was assessed by using the Cochran Q statistic and *I*^*2*^ statistic to represent the proportion of total variation between included studies. Analysis was undertaken on outcomes following LSG, RYGB, SG and intra-abdominal gastric balloon (IAGB), as well as outcomes excluding the IAGB group (Supplementary File 3) as IAGB can be considered an endoscopic procedure rather than surgical. Publication bias and treatment estimate effects were evaluated through funnel plots and conduction of the Egger test. Statistical analyses were carried out using Stata statistical package 12.1 (Stata-Corp., 2011, Stata Statistical Software: Release 12, College Station, TX, USA; StataCorp LP).

### Role of the funding source

Authors A.T.M. and B.D. were awarded Medical Research Council (MRC), Clinical Research Training Fellowships, MR/W015838/1 and MR/V02955X/1, respectively; however the funder had no role in study design, data collection, data analysis, data interpretation, or writing of this report.

## Results

**Fig. 1:**
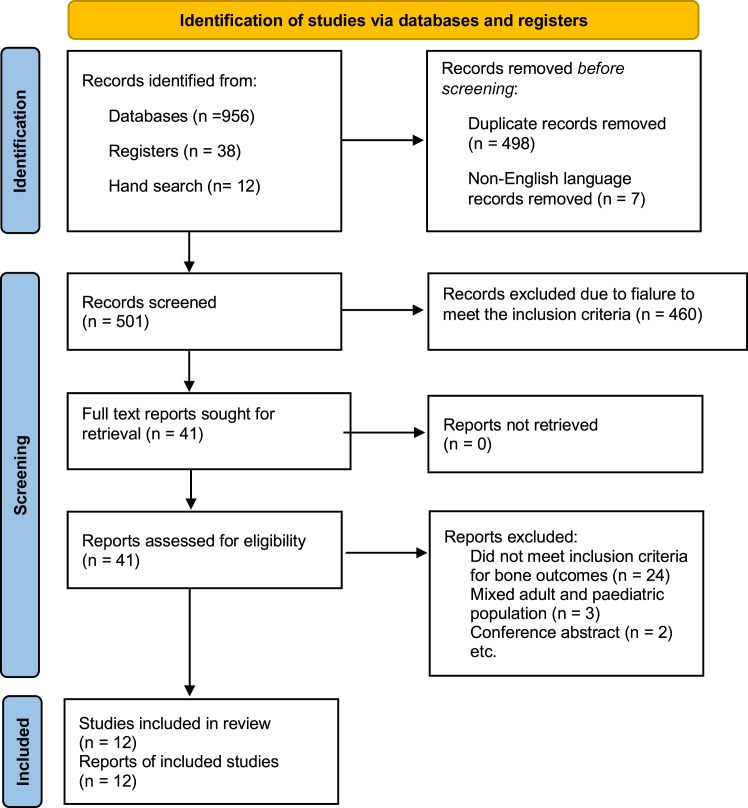
**PRISMA flow chart of search results and studyselection.**

Our review of the literature found 12 studies that were included in the final meta-analysis, out of a total of 1006 identified in the original search, PRISMA flowchart Fig. 1 (PRISMA checklist Supplementary File 4). Nine studies were prospective cohort studies and 2 were retrospective case reviews and 1 was an RCT. The total number of patients was 681 with a mean age of 17 ± 0.57 years, the youngest patient being 8 years, and an overall proportion of 61% being female. Patients underwent either RYGB (n = 216), sleeve gastrectomy (n = 257), gastric band placement (n = 184) or intragastric balloon (n = 24). The average follow up was 16 months (range 8–26 months). Study characteristics and patient demographic data can be found in Table 1. Information regarding post-operative vitamin supplementation and diet restriction can be found in Supplementary File 5.

**Table 1:** Study characteristics & baseline patient demographics for studies included in the meta-analysis.

Author	Publication year	Country	Study design	Sample size	Weight (kg)	Weight SDS	BMI (kg/m^2^)	BMI, SDS	Mean age, range (years)	Age, SDS	Age, range	Lean mass index, Z score	Baseline comorbidity (%)	Gender (% female), total number (n)	Type of bariatric intervention	Length of Follow up (months)	NOS/JADDAD∗	Outcomes collected
Santos et al.^22^	2019	Brazil	Prospective cohort	60			46.1	7.3	17.1	0.8	n/a		Not reported	63.7% (n = 38)	RYGB	12	7	Ca^2+,^ phosphate, 25OH, PTH, Mg, Zinc, ALP
Bredella et al.^23^	2021	USA	Prospective cohort	10	124.1	20.8	43.5	5.6	17.8	2.5	14–21		Not reported	90% (n = 9)	SG	12	7	Ca2+, 25OH, Lumbar BMD
Kaulfers et al.^24^	2010	USA	Retrospective case review	61	154.8	20.4	54.4	6.8	17.3	1.9	13.7–23.5		NALFD (18%), HTN (30%), Depression (33%), OSA (63%), T2DM (11%), PCOS (28%)	83.6% (n = 51)	RYGB	13.9 (2.8–26.8)	6	Lumbar BMD, total body BMC
(A) Misra et al.^25^	2020	USA	Prospective cohort	22	132.7	4.4	47	1.5	18.3	0.5	14–22		T2DM (9%)	72.7% (n = 16)	SG	12	8	Ca^2+,^ phosphate, 25OH, lumbar BMD
Weiner et al.^26^	2020	USA	Retrospective case review	279 (123 with 12-month follow-up)			48	8.8	16.8	1.2	13.4–19.6		Not reported	69.5% (n = 194)	LAGB and SG	12	7	Ca^2+,^25OH, PTH, osteocalcin, CTX
Mitchell et al.^27^	2022	USA	Prospective cohort	30	133.2	25.5	47.1	76.9	18.1	2.1	13–24	2.4	7% (T2DM, 2 SG)	77% (n = 23)	SG	24	8	Ca^2+,^, 25OH, phosphate, PTH, P1NP, CTX, subtotal body and lumbar BMD
Nadler et al.^28^	2009	USA	Prospective cohort	45	135.6	25.8	48.1	6.4	16.1	1.2	14–17		8% OSA	67% (n = 30)	LAGB	12	6	Ca^2+^, 25OH, Mg, zinc, lumbar BMD
Sachdev et al.^29^	2018	UK	Prospective cohort	12	138.5	23.9	46.4	5.6	15.6		n/a		8% OSA, 25% mobility issues, 17% HTN, 58% insulin resistance, 50% dyslipidaemia	58% (n = 7)	IAGB	24	6	Subtotal and lumbar BMD, total BMC
(B) Misra et al.^30^	2020	USA	Prospective cohort	24	134.4	3.7	47.4	1.3	17.8	6.4	14–22	2.6		75% female (n = 18)	SG	12	8	Ca^2+^, phosphate, 25OH, lumbar BMD
De Peppo et al.^31^	2008	Italy	Prospective cohort	12	105.6	15.1	47.9	7.8	18.7	7.1	8–30		50% (4 T2DM, 1 hypothyroidism, 1 scoliosis, 1 HTN)	67% female (n = 8)	IAGB	8	5	Lumbar BMD
Beamish et al. (A)^32^	2016	Sweden	Prospective cohort	22 Male	Male: 145.9	22.5	Male: 46.4	5.6	16.5	1.2	13–18			69% female (n = 52)	RYGB	12 and 24	7	25OH, ALP, osteocalcin
Beamish et al. (B)				50 Female	Female: 123.1	14.5	Female: 44.0	4.8			13–18							P1NP, CTX, lumbar BMD
Jarvholm et al.^33^	2023	Sweden	RCT	25	124.3	16.9	42.9	5	15.6	1.1	13–16	0.6		76% female (n = 19)	RYGB, SG	24	8∗	25OH, subtotal body BMD, PTH

Abbreviations: SDS, standard deviation scores; BMI, body mass index; NALFD, non-alcoholic fatty liver disease; OSA, obstructive sleep apnoea; T2DM, type 2 diabetes mellitus; HTN, hypertension; PCOS, polycystic ovarian disease; RYGB, roux en-Y-gastric bypass; SG, sleeve gastrectomy; IAGB, intra-gastric balloon; LAGB, laparoscopic adjustable gastric band; NOS, Newcastle-Ottowa score, Ca^2+^, calcium; 25OH, 25-hydroxyvitamin D; PTH, parathyroid hormone; Mg, magnesium; ALP, bone alkaline phosphatase; BMD, bone mineral density; CTX, C-terminal telopeptide; P1NP, Procollagen type 1 N propeptide; BMC, bone mineral content; JADDAD∗, modified JADDAD score for RCT.

Quality assessment outcomes of non-randomised studies graded by the NOS ranged from 5 to 8, with the median score being 7, rating the included studies as high quality. The JADDAD score of the RCT was graded as 8 indicating high quality.^33^ The overall NOS and JADDAD scores are presented in Table 1 and study-specific quality scores are presented in Supplementary Files 6a and b. Study heterogeneity ranged from 0 to 99.1%. Publication bias analysis as demonstrated by funnel plots and the Egger test was insignificant for all outcomes apart from 25OHD (p < 0.02) and there was no significant evidence for small studies effect (Supplementary File 7).

**Fig. 2:**
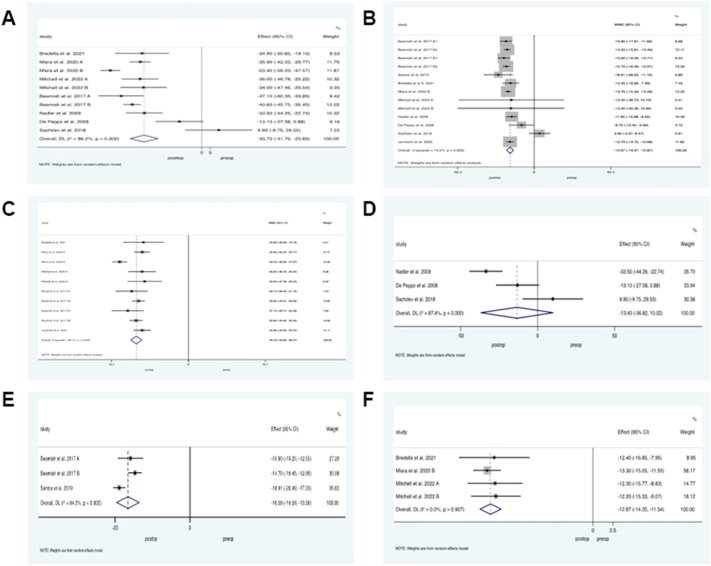
**Weight loss outcomes following paediatric bariatric surgery.** Random effects modelling of meta-analysis data on weight outcomes in paediatric patients before and after bariatric surgery (BS). A) Absolute weight (kg) and B) Body mass index following all BS procedures. C) Weighted mean reduction (kg) following malabsorptive versus D) restrictive BS interventions. E) Weighted mean difference following Roux-en-Y gastric bypass and F) sleeve gastrectomy.

All studies reported significant reduction in absolute weight of 33.7 kg at the study end point (95% CI −41.8 to −25.7, p < 0.0010, *I*^*2*^ = 86%), Fig. 2a, and BMI of −12.67 kg/m^2^ following BS (95% CI −14.86 to −10.9, p < 0.0010, *I*^*2*^ = 74%), Fig. 2b. Analysis based on BS intervention demonstrated, weight loss was greater with SG and RYGB in comparison to IAGB and LAGB, weighted mean reduction of 40 kg (95% CI −44.8 to −36.1, p < 0.0010, *I*^*2*^ = 66%) versus 13.4 kg (95% CI −36.4 to −10, p < 0.001, *I*^*2*^ = 87%), respectively, Fig. 2c and d. On subgroup analysis of RYGB and SG only, RYGB generated greater weight loss over SG, weighted mean BMI reduction of 16.6 kg/m^2^ (95% CI −19.6 to −13.6, p < 0.001, *I*^*2*^ = 84%) versus SG, 12.9 kg/m^2^ (95% CI −14.2 to −11.5, p < 0.0010, *I*^*2*^ = 0%), Fig. 2e and f.

**Fig. 3:**
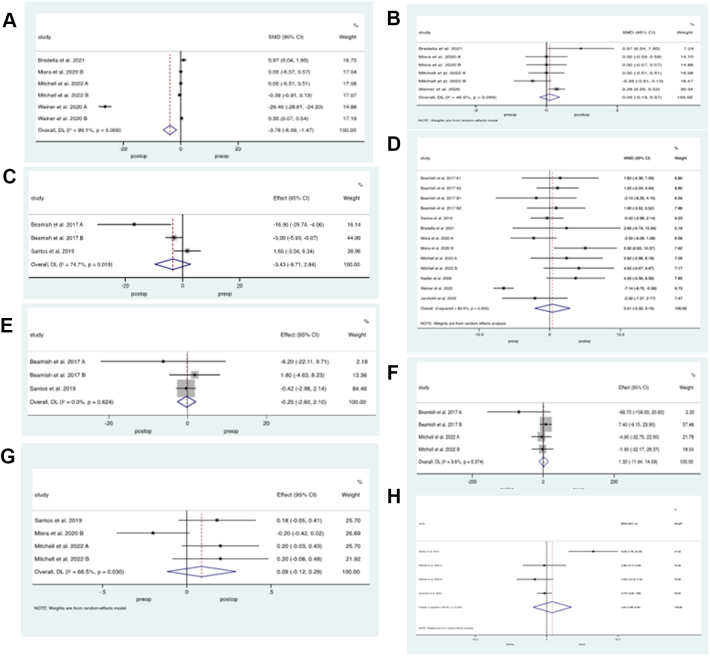
**Bone biochemistry outcomes following Paediatric Bariatric Surgery.** Random effects modelling of meta-analysis data on biochemical bone parameters before and after bariatric surgery (BS). A) Calcium levels from pooled BS interventions and B) from sleeve gastrectomy (SG) only, C) bone alkaline phosphatase and D) 25-hydroxyvitamin D levels from combined BS procedures and from, E) SG and Roux-en-Y gastric bypass only, F) procollagen type 1 N propeptide levels, G) phosphate and H) parathyroid levels following BS.

Regarding bone biochemistry, four studies^23^^,^^26^^,^^27^^,^^34^ with a total of 744 patients who underwent SG or RYGB evaluated total calcium levels before and after BS. Pooled results demonstrated lower calcium following BS, standardized mean reduction of −3.8 mg/dl (95% CI −6.1 to −1.5, p = 0.001, *I*^*2*^ = 99%), Fig. 3a. Of those patients, the majority (n = 510) underwent SG and on analysis of this group only, results did not demonstrate any difference in calcium levels before or after surgery, standardized mean difference 0.1 mg/dl (95% CI −0.19 to 0.37, p = 0.52, *I*^*2*^ = 46%), Fig. 3b.

Three studies^22^^,^^28^^,^^32^ with a total of 264 patients reported ALP before and after RYGB. Overall, there was no change in ALP following BS, weighted mean difference of −3.431 U/l (95% CI −9.71 to 2.84, p = 0.23, *I*^*2*^ = 75%), Fig. 3c.

Eleven studies^22^^,^^23^^,^^25^, ^26^, ^27^, ^28^^,^^30^^,^^32^^,^^33^ with a total of 553 patients evaluated 25OHD levels pre- and post-BS. For all studies combined, there was no difference in 25OHD, 0.41 ng/ml (95% CI −2.3 to 3.1, p = 0.77, *I*^*2*^ = 84%), Fig. 3d. Subgroup analysis by studies only performing SG (n = 452) or RYGB (n = 264) reported no difference in 25OHD levels between the groups following surgery, −0.25 ng/ml (95% CI −2.6 to 2.1, p = 0.62, *I*^*2*^ = 0%), respectively Fig. 3e. Four studies^27^^,^^32^ with a total of 262 patients who underwent SG or RYGB evaluated P1NP levels, demonstrating no difference following BS, 1.32 ng/ml (95% CI −11.9 to 14.6, p = 0.37, *I*^*2*^ = 3.6%), Fig. 3f. Four studies with a total of 286 patients who underwent SG or RYGB evaluated phosphate levels before and after surgery. Results did not show any difference in this group, weighted mean difference of 0.09 mg/dl (95% CI −0.12 to 0.3, p = 0.4, *I*^*2*^ = 67%), Fig. 3g. Three studies^22^^,^^27^^,^^30^ with a total of 169 patients who underwent SG or RYGB evaluated PTH levels before and after surgery demonstrating no significant difference in levels, weighted mean difference 1.9 pg/ml (95% CI −4.6 to 8.4, p = 0.56, *I*^*2*^ = 81%), Fig. 3h.

**Fig. 4:**
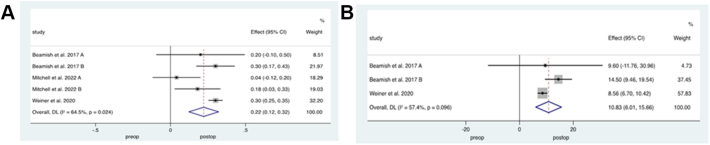
**Bone resorptive markers following Paediatric Bariatric Surgery.** Random effects modelling of meta-analysis data on markers of bone resorption before and after bariatric surgery (BS). Weighted mean difference in A) C-terminal telopeptide and B) Osteocalcin following bariatric surgery.

Markers of bone resorption were evaluated by measuring CTX and osteocalcin. Three^26^^,^^27^^,^^32^ studies with a total of 820 patients who underwent SG or RYGB evaluated CTX levels before and after surgery. Meta-analysis of outcomes revealed significantly higher levels following BS, weighted mean difference 0.22 ng/ml (95% CI 0.12–0.32, p < 0.001, *I*^*2*^ = 66%), Fig. 4a. Three studies^26^^,^^32^ with a total of 702 patients who underwent either SG or RYGB evaluated osteocalcin levels, demonstrating significantly increased levels following BS, weighted mean difference 10.83 ng/ml (95% CI 6.01–15.7, p = 0.00, *I*^*2*^ = 57%), Fig. 4b.

**Fig. 5:**
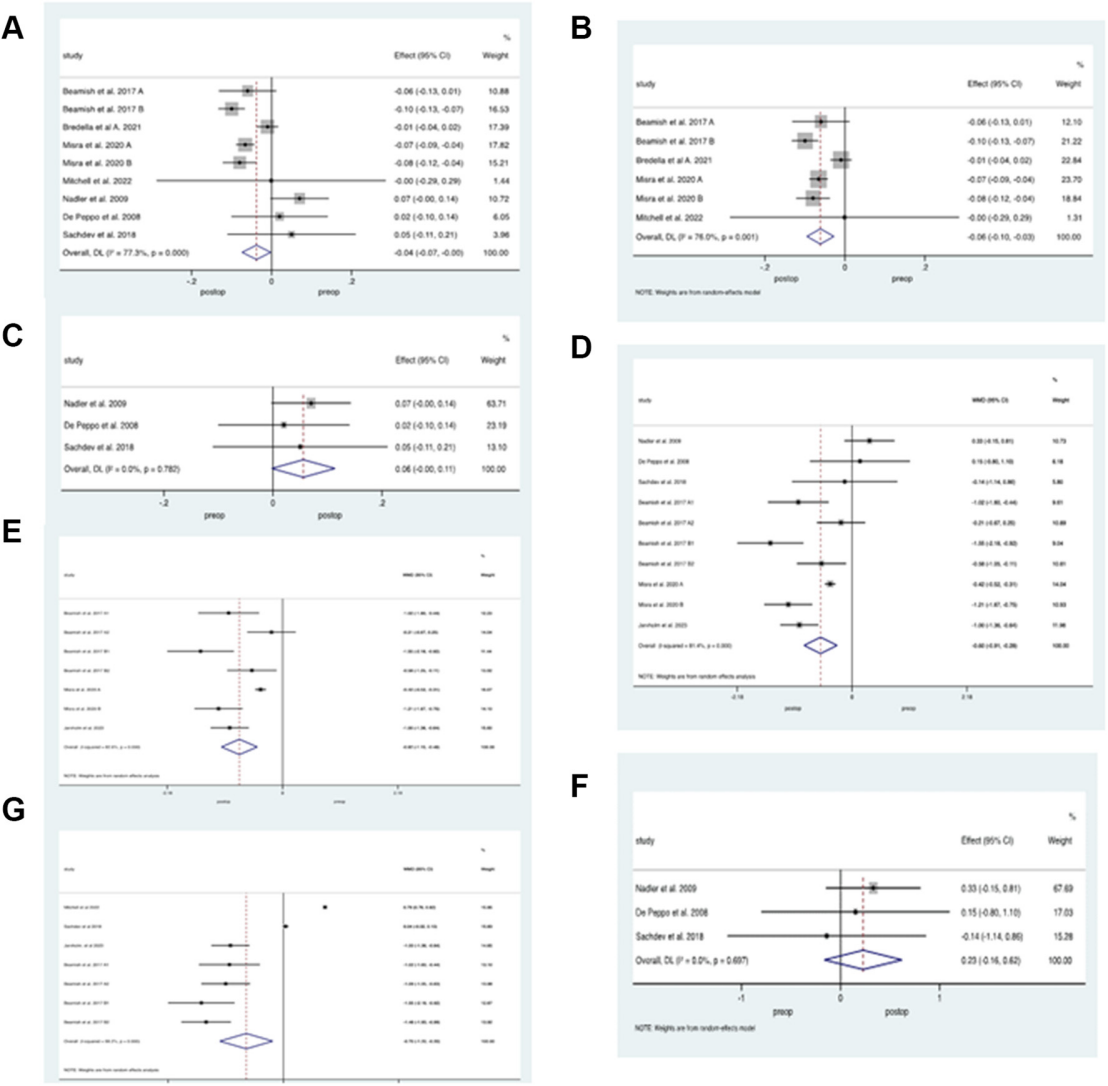
**Radiological markers of Bone Health following Paediatric Bariatric Surgery.** Random effects modelling of meta-analysis data on radiological evaluation of bone health in paediatric patients before and after bariatric surgery (BS). A) Lumbar bone mineral density (BMD) from all procedures combined, B) BMD from malabsorptive BS and C) restrictive BS interventions, D) Z score from pooled BS, E) Z score from malabsorptive BS, F) restrictive BS interventions and G) subtotal bone mineral density.

Radiological evaluation of bone health was determined by measuring lumbar BMD, Z scores and subtotal body bone mineral density (sbBMD). Eight studies^23^^,^^25^^,^^27^, ^28^, ^29^, ^30^, ^31^, ^32^ with a total of 454 patients evaluated lumbar BMD levels before and after BS. Results demonstrated significantly lower BMD following pooled BS procedures, weighted mean reduction of −0.04 g/cm^2^ (95% CI −0.07 to 0.002, p = 0.04, *I*^*2*^ = 77%), Fig. 5a. Subgroup analysis by mixed restrictive and malabsorptive procedures (SG and RYGB, n = 316 patients) versus restrictive procedures only (IAGB or LAGB, n = 138 patients) demonstrated a significant reduction in BMD in patients who underwent SG or RYGB only, standardized weighted reduction of −0.06 g/cm^2^ (95% CI −0.03 to −0.1, p < 0.001, *I*^*2*^ = 76%), Fig. 5b. There was no difference in lumbar BMD in patients who underwent IAGB or LAGB, weighted mean difference of 0.06 (95% CI −002 to 0.114, p = 0.06, *I*^*2*^ = 0%), Fig. 5c.

Seven studies^25^^,^^28^, ^29^, ^30^, ^31^, ^32^, ^33^ with a total of 284 participants reported Z scores before and after BS. There was a significant reduction in Z score with pooled BS procedures, weighted mean reduction of −0.6 (95% CI −0.9 to −0.3, p < 0.0010, *I*^*2*^ = 81%), Fig. 5d. On subgroup analysis, by procedure, there was significantly lower Z score with SG or RYGB, weighted mean reduction of −0.82 (95% CI −1.6 to −0.5, p < 0.0010, *I*^*2*^ = 83%), Fig. 5e, but no difference with IAGB or LAGB, weighted mean difference of 0.23 (95% CI −0.16 to 0.62, p = 0.25, *I*^*2*^ = 0%), Fig. 5f.

Four studies^27^^,^^32^^,^^33^^,^^35^ with a total of 139 patients evaluated sbBMD following BS, this refers to the measurement of BMD in multiple regions or a significant portion of the body rather than focusing solely on a particular region (e.g., lumbar) to give a more holistic picture of bone health. Results reported a significant reduction in BMD following surgery, weight mean reduction of −0.7 g/cm^2^ (95% CI −1.2 to −0.2, p < 0.0016, *I*^*2*^ = 99%), Fig. 5g.

## Discussion

This is the first systematic review and meta-analysis to evaluate the effects of BS in the paediatric population on bone outcomes. The principal findings are, BS is highly effective at achieving significant weight loss however, SG and RYGB are associated with poorer markers of bone health in the medium-term. In addition, untreated micronutrient deficiencies in childhood are associated with other systemic illnesses such as autoimmune disorders, cognitive impairment and increased susceptibility to infections.^36^, ^37^, ^38^ Acknowledging these risks post paediatric BS and treating them promptly is fundamental given the increase in global childhood obesity and the rising application of BS as a solution to this problem.

Analysis of bone outcomes based on the type of BS intervention demonstrated that patients who underwent SG and RYGB had significantly lower calcium, subtotal body and lumbar BMD and Z scores with higher bone resorption markers in comparison to their preoperative values. When all BS procedures were pooled, some of these differences were negated which suggests the effect is primarily driven by malabsorptive procedures rather than restrictive ones. This finding was further corroborated in the subgroup analysis that excluded patients who had IAGB. Our findings did not show a difference in bone ALP, 25OHD, PTH, P1NP or phosphate. These findings may be explained by several reasons. From a mechanistic point of view, the intake and absorption of nutrients, particularly bone essential 25OHD and calcium, are directly affected by the anatomical disruption of the upper gastrointestinal tract caused by BS. For RYGB, a small gastric pouch is created with bypassing of the duodenum and majority of the jejunum, however, most dietary calcium is absorbed via active transporters in these bypassed segments.^39^ In addition, the smaller gastric pouch size, produces less gastric acid and enzymatic secretions which adversely impacts nutrient digestion and solubility contributing to malabsorption.^40^ Nutrients such as 25OHD are fat-soluble and require bile and digestive enzymes for optimal absorption therefore patients who have undergone RYGB may develop vitamin and micronutrient deficiencies due to a culmination of malabsorption, bypassed bowel segments and reduced digestion.^41^ The malabsorptive picture may be further exacerbated by perturbations in the secretion of bone essential hormones. Reports from adult studies suggest secretion of hormones such as PTH and consequently calcium are altered following RYGB as well as differences in calcitriol (active form of 25OHD), and fibroblast growth factor.^42^ For SG, although primarily considered a restrictive procedure, there has been a shift towards recognising the risk of hypovitaminosis and consequently the need for life long nutritional supplementation. Whilst the primary effects of SG are mediated through a reduction in gastric size, this generates a concurrent reduction in gastric acid secretion and absorption of intrinsic factor that adversely impacts nutritional balance and vitamin B12 and iron absorption.^43^, ^44^, ^45^ As a result, it is now advised that patients are placed on mandatory vitamin and nutrient supplementation following SG as well as RYGB.^46^ Currently, there is lack of consensus regarding the optimal post-operative nutritional strategy for paediatric patients undergoing BS however this is crucial to consider based on results generated in our study as well as extrapolation of data from adults undergoing SG.^46^, ^47^, ^48^

We did not identify any differences in 25OH, PTH, bone ALP or any trace mineral in this study, which may reflect compensation in bone turnover and remodelling to maintain skeletal homeostasis or increased renal absorption of some micronutrients as BS is paradoxically known to have renoprotective effects.^49^^,^^50^ The reduced levels of vitamins and minerals can trigger a negative feedback loop stimulating greater bone resorption through increased osteoclast activity and bone breakdown, an effect which was seen in this review.^51^ Such metabolic alterations can explain why patients with a history of BS are at increased risk of poorer bone outcomes however it is important to validate these hypotheses through mechanistic evaluation. Specifically, for 25OHD, we may not have detected a difference here as the included papers were predominantly from Northern European or American countries reflecting Caucasian populations where the prevalence of vitamin D deficiency is less in comparison to countries with Asian, African, or non-white ethnic minority populations.^52^^,^^53^

Aside from the malabsorptive explanations, adverse bone outcomes associated with BS may also be attributed to bone loading mechanisms. Functionally, weight and its gain and loss are important components for stimulating bone growth. Increased fat mass up to a healthy range has traditionally been considered as ‘bone-protective’ since mechanical loading is associated with higher BMD through proliferation of osteoblasts and reduction of bone apoptosis.^54^^,^^55^ Healthy bones respond to mechanical stress up to a reasonable limit by remodelling, and a decrease in mechanical loading through weight loss may lead to bone loss.^56^^,^^57^ This effect may be pronounced in weight-bearing bones such as the lumbar region that was evaluated in this study and may explain our findings as the procedures that generate the most weight loss have lower BMD and Z scores. However, this may not be the sole mechanism for bone loss following mechanical unloading. Diniz-Souza et al. evaluated BMD in 21 patients undergoing RYGB demonstrating gravitational loading only decreased during the first month after surgery correlating to weight loss but stabilised after that. These changes did not fully explain BS-induced bone loss and the effects seen may be associated with other physiological aspects, such as fat and lean mass loss, rather than solely from gravitational loading decrease.^58^

There were no changes in bone parameters seen with IAGB or LAGB. This may be because these interventions are not as intricately associated with post-operative nutrient deficiencies or considered to produce the degree of severity of malabsorption as RYGB. IAGB or LAGB utilise restrictive mechanisms by reducing gastric volume and hence food intake to generate weight loss so avoid directly interfering with intestinal absorption of vitamins.

It is important to leverage the medium-term risks of childhood bone loss against the overall health benefits of weight loss. Weight loss is known to be a protective factor for osteoporosis,^59^ and it is well regarded that weight loss has positive wider effects on systematic metabolism and hormonal profiles. BS is an effective treatment for the remission of obesity-associated medical comorbidities in adults and this positive effect has also been reported in children. Inge et al. demonstrated higher rates of remission of hypertension and diabetes in adolescents undergoing BS in comparison to adults, and an improvement in physical and psychosocial health that is associated with weight loss during childhood.^60^^,^^61^ Furthermore, obesity is known to be a damaging lifestyle factor that negatively impacts bone accrual during adolescence, remodelling and bone mass.^16^ It may be that although BS induced weight loss negatively impacts bone health in the medium-term, this may be less harmful than the overall long-term effects of untreated obesity. In order to answer this important question, long-term follow up of adequately age and weight matched control groups is required.

Although the seminal findings of this review are mirrored in the adult population, it is important to acknowledge some limitations of this study. The youngest patient in this group was 8 years old which indicates that currently, the majority of paediatric BS occurs during adolescence, not in the younger childhood years so the results of this study may not be applicable to the younger age group. Aside from a paper published by Alqahtani et al. who reported BS outcomes on a cohort of 2504 participants the youngest being 5 years,^11^ this age range is typical of the broader literature base in this field. There is also no long-term data beyond 2 years. Follow up over a longer time is imperative in children to evaluate overall trends in bone health and whether the reported results translate to a clinically meaningful increase in fracture risk or development of oesteomalacia. It is also important to determine whether the decline in bone health is transient as adolescents have greater elasticity for the reversal of comorbidities so bone mass may be accrued later in life and bone loss may be reversed in the long term.^62^ It is generally agreed that peak bone mineral density is attained by the age of 30, with men continuing to accrue bone mass slightly later than women,^63^ therefore earlier intervention with BS may potentially give more opportunity for the recovery of bone mass over time, a question that can only be answered with long-term data. Studies in adolescent groups who had detrimental impacts on bone health during critical periods of growth have demonstrated the possibility of recovery and a ‘catch-up’ effect.^64^^,^^65^ Perhaps the greatest benefit of long-term data would be to determine the risk and benefit in these patients in terms of the proportion of patients having significant weight regain versus the number of patients that develop premature osteopenia or osteoporosis as a result of undergoing BS. This would determine, in the long term, if the benefits of undergoing BS in childhood outweigh the risks of bone loss or whether it would be preferable to postpone BS for some years until reaching adulthood.

Several studies have specified whether post-operative vitamin or nutritional supplementation was provided however it is unclear whether this was adhered to.^33^ It is generally regarded that adherence to medication is poorer in children and adolescents thus implementation of life-long of vitamin supplementation may be challenging,^66^ something that was reported by Santos et al. who required their participants to show the containers of their supplements during follow-up.^22^^,^^66^ Furthermore, it has been reported that rapid changes in soft tissue mass may affect the accuracy of BMD DEXA measurements. Fat layering may introduce error in bone density readings and decrease the reproducibility of BMD spine and hip parameters in humans.^67^ To mitigate for these potential issues, a more uniform measurement for patients having significant changes in their body mass would be to use quantitative computer tomography (qCT) BMD,^68^ which only Mitchell et al. reported^27^ in our included studies.

The wider limitations of paediatric BS are applicable to this review, being small sample size of studies, heterogeneity within the studies pooled indicated by the range of *I*^*2*^ statistics and paucity of data on re-intervention or re-operative rates. This information is essential for clinicians, policy makers and patients, so the long-term hazards of undergoing BS during childhood can be comprehensively evaluated.

The methodological strengths of this review include the quality of the included studies as measured by the NOS and JADDAD scores, the study design of most articles was prospective and pooling of the data by nature of performing a meta-analysis facilitated the generation of a large sample size to evaluate outcomes in this niche field.

In conclusion, BS is highly effective at achieving weight loss in paediatric patients however SG and RYGB is associated with poorer markers of bone health. Current recommendations from commissioning bodies, suggest adolescent bariatric surgery should be considered in ‘exceptional circumstances’,^69^ however the case for this may be expanded given the overwhelming positive impact it has on weight specific and generalised health outcomes. It is however important to recognise the vulnerabilities of this group in developing micronutrient deficiencies translating to medium and long-term health risks after BS. Considering the landscape of weight loss strategies is rapidly changing due to licensed medical therapies, this may affect the role of BS in paediatric patients and will be of interest to follow over time. This review demonstrates that bone health needs careful attention and long-term follow up as it is unclear whether the short-term bone loss translates to poorer overall clinical outcomes in paediatric patients undergoing BS.

## Contributors

Conceptualization AM, BD, and MF; AM and BD performed literature search, article screening, data extraction, analyses and manuscript write up. MF and KS performed a secondary review of articles and directly accessed and verified the underlying data reported. AM, BD, and MF original draft preparation, preparation of figures. AM and BD original manuscript draft. KS, MFA, MF, and HA writing—review and editing. MFA, MF, and HA supervision. All authors have read and agreed to the published version of the manuscript. All authors confirm they had full access to all the data in the study and accept responsibility to submit for publication.

## Data sharing statement

The data that support the findings of this study are available from the corresponding author, [ATM], upon reasonable request.

## Declaration of interests

The authors declare no conflicts of interests.
