# Supplementary material for: Bone health following paediatric and adolescent bariatric surgery: a systematic review and meta-analysis
Source: eClinicalMedicine. 2024 Feb 2;69:102462. doi: 10.1016/j.eclinm.2024.102462 (PMC10850131; doi:10.1016/j.eclinm.2024.102462)

**Supplementary Material**

Contents

[Supplementary file 1: Search strategy 2](#_Toc154573937)

[Supplementary file 2: AMSTAR Checklist 4](#_Toc154573938)

[Supplementary File 3: Forrest Plots on Data Outcomes excluding the Intragastric Balloon Cohort 5](#_Toc154573939)

[Supplementary file 4: PRISMA 2020 Main Checklist 6](#_Toc154573940)

[PRIMSA Abstract Checklist 10](#_Toc154573941)

[Supplementary file 5: Information regarding post operative nutrition, exercise and diet 12](#_Toc154573942)

[Supplementary file 6a: Newcastle Ottowa Score for nonrandomized studies 13](#_Toc154573943)

[Supplementary file 6b: Modified JADDAD score for Randomized Controlled Trials 15](#_Toc154573944)

[Supplementary file 7: Funnel Plots & Eggers test for publication bias 16](#_Toc154573945)

# Supplementary file 1: Search strategy

**Developing the search strategy**

The search strategy was required to capture all published evidence reporting bone outcome measures in paediatric patients who underwent bariatric surgery. Review articles, conference abstracts, and animal and cell studies were excluded. The search strategy took account of the variability in the way authors describe their research and indexers index research with Medical Subject Headings (MeSH). All variations in spelling including truncated search terms using wild card characters and the “related articles” function were used in combination with the Boolean operators AND OR.

The following databases were searched:

• Ovid MEDLINE (R) 1946 to 2023 September 28;

• Ovid Embase 1974 to 2023 September 28;

• Cochrane library from inception to 2023 September 28

Records identified from each electronic and regional database were downloaded into a separate Endnote bibliographic database, which were then combined to generate a single large Endnote bibliography containing all the records identified from all the databases in which duplicate records were removed.

Final search strategy

The final search strategy and results for the MEDLINE search is shown below:

Box 1 Key:

Exp Explodes a MeSH to capture more specific MeSH.

/ Indicates that the search term is a Medical Subject Heading (MeSH).

AND Achieves a Boolean AND Combination.

OR Achieves a Boolean OR Combination.

.mp. Searches for the term expressed in the title, original title, abstract, and subject heading.

* Truncation operator, searches for words beginning with the stem, e.g. epidemiol* retrieves epidemiology, epidemiological and epidemiologic.

**Medline search**

Ovid MEDLINE(R) ALL <1946 to September 28, 2023>

| 1 | exp Child/ or child.mp. | 2323540 |
| --- | --- | --- |
| 2 | exp Adolescent/ or exp Adolescent Health/ or adolescent.mp. | 2232479 |
| 3 | exp Infant/ or exp Child, Preschool/ or paediatric.mp. or Pediatrics/ or exp Adolescent/ | 3399877 |
| 4 | 1 or 2 or 3 | 4082692 |
| 5 | Pediatric Obesity/ or Obesity Management/ or exp Obesity/ or obesity.mp. or exp Obesity, Morbid/ | 389388 |
| 6 | childhood obesity.mp. or exp Pediatric Obesity/ | 19927 |
| 7 | overweight.mp. or exp Obesity/ or exp Male/ or exp Overweight/ or Body Weight/ | 9491801 |
| 8 | fat.mp. | 317216 |
| 9 | exp Body Mass Index/ or high bmi.mp. | 148032 |
| 10 | 5 or 6 or 7 or 8 or 9 | 9716857 |
| 11 | 4 and 10 | 2430969 |
| 12 | exp Gastroplasty/ or bariatric surgery.mp. or exp Bariatric Surgery/ or exp Gastric Bypass/ | 38436 |
| 13 | metabolic surgery.mp. | 1425 |
| 14 | weight loss surgery.mp. | 1039 |
| 15 | exp Weight Loss/ or exp Gastrectomy/ or weight loss procedure.mp. | 86567 |
| 16 | gastric balloon.mp. or exp Gastric Balloon/ | 921 |
| 17 | gastric band.mp. | 1255 |
| 18 | 12 or 13 or 14 or 15 or 16 or 17 | 114032 |
| 19 | 4 and 11 and 18 | 9738 |
| 20 | paediatric bariatric surgery.mp. | 4 |
| 21 | child.mp. or exp child/ | 2323540 |
| 22 | paediatric.mp. or exp pediatrics/ | 129011 |
| 23 | teenager.mp. or exp adolescent/ | 2195159 |
| 24 | young adult.mp. or exp young adult/ | 1024998 |
| 25 | pediatric.mp. or exp pediatrics/ | 400114 |
| 26 | 21 or 22 or 23 or 24 or 25 | 4078612 |
| 27 | exp obesity/ or exp childhood obesity/ or obesity.mp. or exp diabetic obesity/ or exp morbid obesity/ or exp obesity management/ | 398375 |
| 28 | exp fat/ or fat.mp. or exp body fat/ | 365819 |
| 29 | overweight.mp. | 89250 |
| 30 | 27 or 28 or 29 | 698760 |
| 31 | 26 and 30 | 137114 |
| 32 | bariatric surgery.mp. or exp bariatric surgery/ | 38436 |
| 33 | metabolic surgery.mp. or bariatric surgery/ | 14819 |
| 34 | bariatric operation.mp. | 315 |
| 35 | bariatric intervention.mp. | 73 |
| 36 | 32 or 33 or 34 or 35 | 38705 |
| 37 | 31 and 36 | 5211 |

# Supplementary file 2: AMSTAR Checklist

Please see attached PDF

# Supplementary File 3: Forrest Plots on Data Outcomes excluding the Intragastric Balloon Cohort


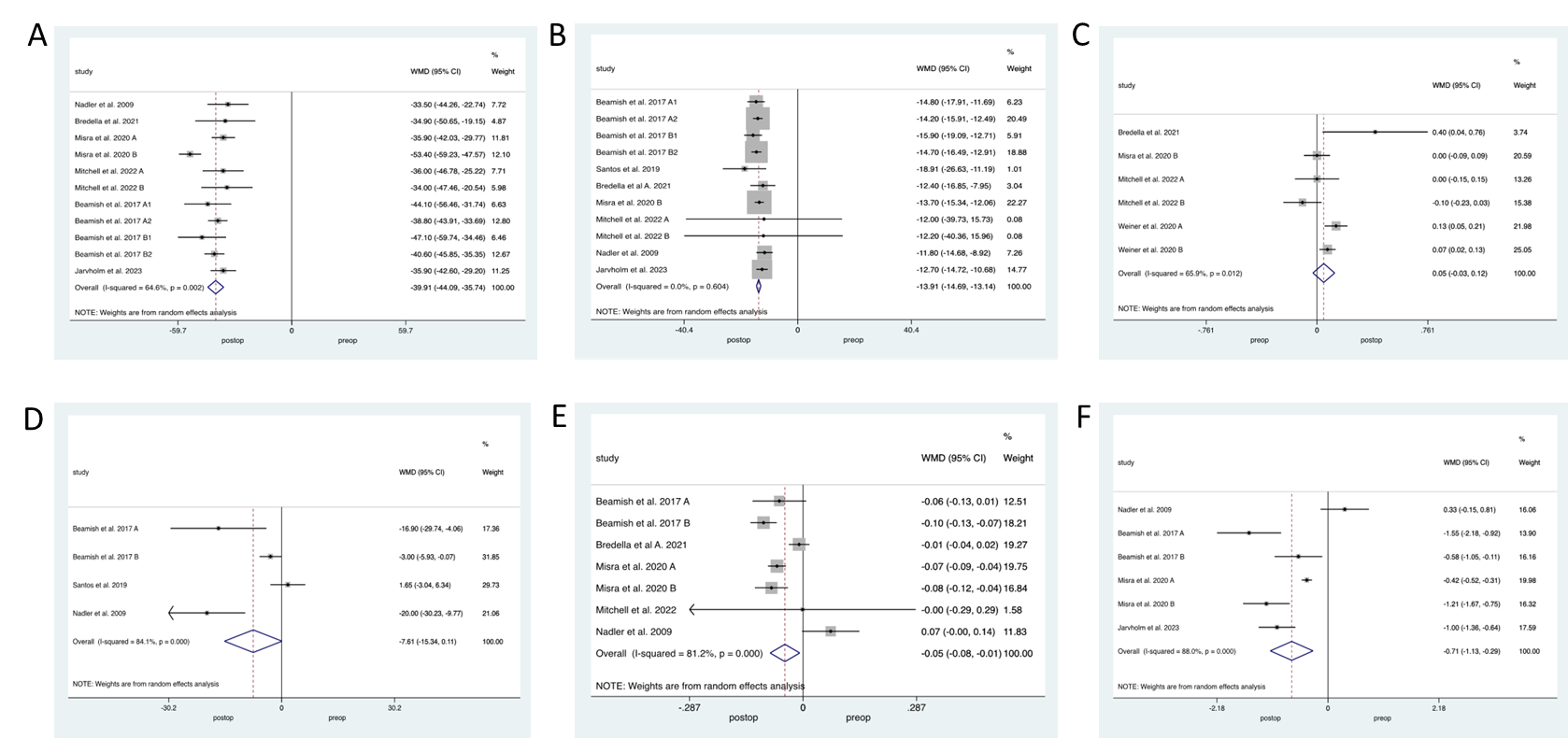


Random effects modelling of meta- analysis data from LSG, RYGB and GB only on A) Absolute weight (kg), -39.91 kg (95% CI -35.74 to -44.09, p=0.002, *I^2^*=64.6%, B) Body mass index, -13.92 kg/m^2^ (95% CI -13.14 to -14.69, p=0.6, *I^2^ 0%*), ), C) Calcium 0.05 mg/dl (95% CI -0.03 to 0.12, p= 0.01, *I^2^*=65.9%), D) ALP -7.61 U/l (95% CI -15.34 to 0.11, p= 0.001, *I^2^* 84.1%), E) BMD all studies -0.05 (95% CI -0.08 to -0.01, p= 0.001, *I^2^* 81.2%), F) Z score, -0.71 (95% CI -0.29 to -1.13, p=0.001, *I^2^*= 88%).

# Supplementary file 4: PRISMA 2020 Main Checklist

| **Topic** | **No.** | **Item** | **Location where item is reported** |
| --- | --- | --- | --- |
| **TITLE** |  |  |  |
| **Title** | 1 | Identify the report as a systematic review. | 1 |
| **ABSTRACT** |  |  |  |
| **Abstract** | 2 | See the PRISMA 2020 for Abstracts checklist | 2-3 |
| **INTRODUCTION** |  |  |  |
| **Rationale** | 3 | Describe the rationale for the review in the context of existing knowledge. | 4 |
| **Objectives** | 4 | Provide an explicit statement of the objective(s) or question(s) the review addresses. | 4, 8 |
| **METHODS** |  |  |  |
| **Eligibility criteria** | 5 | Specify the inclusion and exclusion criteria for the review and how studies were grouped for the syntheses. | 8-9 |
| **Information sources** | 6 | Specify all databases, registers, websites, organisations, reference lists and other sources searched or consulted to identify studies. Specify the date when each source was last searched or consulted. | 8 |
| **Search strategy** | 7 | Present the full search strategies for all databases, registers and websites, including any filters and limits used. | Supplementary file 1 |
| **Selection process** | 8 | Specify the methods used to decide whether a study met the inclusion criteria of the review, including how many reviewers screened each record and each report retrieved, whether they worked independently, and if applicable, details of automation tools used in the process. | 8-9 |
| **Data collection process** | 9 | Specify the methods used to collect data from reports, including how many reviewers collected data from each report, whether they worked independently, any processes for obtaining or confirming data from study investigators, and if applicable, details of automation tools used in the process. | 8-9 |
| **Data items** | 10a | List and define all outcomes for which data were sought. Specify whether all results that were compatible with each outcome domain in each study were sought (e.g. for all measures, time points, analyses), and if not, the methods used to decide which results to collect. | 10 |
|  | 10b | List and define all other variables for which data were sought (e.g. participant and intervention characteristics, funding sources). Describe any assumptions made about any missing or unclear information. | 8 |
| **Study risk of bias assessment** | 11 | Specify the methods used to assess risk of bias in the included studies, including details of the tool(s) used, how many reviewers assessed each study and whether they worked independently, and if applicable, details of automation tools used in the process. | 9 |
| **Effect measures** | 12 | Specify for each outcome the effect measure(s) (e.g. risk ratio, mean difference) used in the synthesis or presentation of results. | 10 |
| **Synthesis methods** | 13a | Describe the processes used to decide which studies were eligible for each synthesis (e.g. tabulating the study intervention characteristics and comparing against the planned groups for each synthesis (item 5)). | 10 |
|  | 13b | Describe any methods required to prepare the data for presentation or synthesis, such as handling of missing summary statistics, or data conversions. | 10 |
|  | 13c | Describe any methods used to tabulate or visually display results of individual studies and syntheses. | n/a |
|  | 13d | Describe any methods used to synthesize results and provide a rationale for the choice(s). If meta-analysis was performed, describe the model(s), method(s) to identify the presence and extent of statistical heterogeneity, and software package(s) used. | 10 |
|  | 13e | Describe any methods used to explore possible causes of heterogeneity among study results (e.g. subgroup analysis, meta-regression). | 10 |
|  | 13f | Describe any sensitivity analyses conducted to assess robustness of the synthesized results. | 10 |
| **Reporting bias assessment** | 14 | Describe any methods used to assess risk of bias due to missing results in a synthesis (arising from reporting biases). | 9-10 |
| **Certainty assessment** | 15 | Describe any methods used to assess certainty (or confidence) in the body of evidence for an outcome. | 9-10 |
| **RESULTS** |  |  |  |
| **Study selection** | 16a | Describe the results of the search and selection process, from the number of records identified in the search to the number of studies included in the review, ideally using a flow diagram. | 10-12 |
|  | 16b | Cite studies that might appear to meet the inclusion criteria, but which were excluded, and explain why they were excluded. | 10 |
| **Study characteristics** | 17 | Cite each included study and present its characteristics. | 12-14 |
| **Risk of bias in studies** | 18 | Present assessments of risk of bias for each included study. | 12-14 |
| **Results of individual studies** | 19 | For all outcomes, present, for each study: (a) summary statistics for each group (where appropriate) and (b) an effect estimate and its precision (e.g. confidence/credible interval), ideally using structured tables or plots. | 12-14 |
| **Results of syntheses** | 20a | For each synthesis, briefly summarise the characteristics and risk of bias among contributing studies. | 12-14 |
|  | 20b | Present results of all statistical syntheses conducted. If meta-analysis was done, present for each the summary estimate and its precision (e.g. confidence/credible interval) and measures of statistical heterogeneity. If comparing groups, describe the direction of the effect. | 12-14 |
|  | 20c | Present results of all investigations of possible causes of heterogeneity among study results. | 12-14, supplementary file 7 |
|  | 20d | Present results of all sensitivity analyses conducted to assess the robustness of the synthesized results. | 12-14 |
| **Reporting biases** | 21 | Present assessments of risk of bias due to missing results (arising from reporting biases) for each synthesis assessed. | n/a |
| **Certainty of evidence** | 22 | Present assessments of certainty (or confidence) in the body of evidence for each outcome assessed. | 12-14 |
| **DISCUSSION** |  |  |  |
| **Discussion** | 23a | Provide a general interpretation of the results in the context of other evidence. | 14-15 |
|  | 23b | Discuss any limitations of the evidence included in the review. | 18-19 |
|  | 23c | Discuss any limitations of the review processes used. | 19 |
|  | 23d | Discuss implications of the results for practice, policy, and future research. | 17-19 |
| **OTHER INFORMATION** |  |  |  |
| **Registration and protocol** | 24a | Provide registration information for the review, including register name and registration number, or state that the review was not registered. | 8 |
|  | 24b | Indicate where the review protocol can be accessed, or state that a protocol was not prepared. | 8 |
|  | 24c | Describe and explain any amendments to information provided at registration or in the protocol. | n/a |
| **Support** | 25 | Describe sources of financial or non-financial support for the review, and the role of the funders or sponsors in the review. | 10, 20 |
| **Competing interests** | 26 | Declare any competing interests of review authors. | 20 |
| **Availability of data, code and other materials** | 27 | Report which of the following are publicly available and where they can be found: template data collection forms; data extracted from included studies; data used for all analyses; analytic code; any other materials used in the review. | 20 |

#####

## PRIMSA Abstract Checklist

| **Topic** | **No.** | **Item** | **Reported?** |
| --- | --- | --- | --- |
| **TITLE** |  |  |  |
| **Title** | 1 | Identify the report as a systematic review. | Yes |
| **BACKGROUND** |  |  |  |
| **Objectives** | 2 | Provide an explicit statement of the main objective(s) or question(s) the review addresses. | Yes |
| **METHODS** |  |  |  |
| **Eligibility criteria** | 3 | Specify the inclusion and exclusion criteria for the review. | Yes |
| **Information sources** | 4 | Specify the information sources (e.g. databases, registers) used to identify studies and the date when each was last searched. | Yes |
| **Risk of bias** | 5 | Specify the methods used to assess risk of bias in the included studies. | No |
| **Synthesis of results** | 6 | Specify the methods used to present and synthesize results. | Yes |
| **RESULTS** |  |  |  |
| **Included studies** | 7 | Give the total number of included studies and participants and summarise relevant characteristics of studies. | Yes |
| **Synthesis of results** | 8 | Present results for main outcomes, preferably indicating the number of included studies and participants for each. If meta-analysis was done, report the summary estimate and confidence/credible interval. If comparing groups, indicate the direction of the effect (i.e. which group is favoured). | Yes |
| **DISCUSSION** |  |  |  |
| **Limitations of evidence** | 9 | Provide a brief summary of the limitations of the evidence included in the review (e.g. study risk of bias, inconsistency and imprecision). | Yes |
| **Interpretation** | 10 | Provide a general interpretation of the results and important implications. | Yes |
| **OTHER** |  |  |  |
| **Funding** | 11 | Specify the primary source of funding for the review. | Yes |
| **Registration** | 12 | Provide the register name and registration number. | Yes |

*From:* Page MJ, McKenzie JE, Bossuyt PM, Boutron I, Hoffmann TC, Mulrow CD, et al. The PRISMA 2020 statement: an updated guideline for reporting systematic reviews. MetaArXiv. 2020, September 14. DOI: 10.31222/osf.io/v7gm2. For more information, visit: [www.prisma-statement.org](file:///C:\Users\Anuja\Downloads\www.prisma-statement.org)

# Supplementary file 5: Information regarding post operative nutrition, exercise and diet

| Authors | Vitamin supplementation | Exercise | Nutrition information |
| --- | --- | --- | --- |
| Misra, et al 2021 | 1200mg elemental calcium daily  800 IU vitamin D daily | Exercise counselling provided by research centre | Exercise counselling provided by research centre |
| Misra et al 2020 | 1200mg elemental calcium daily  800 IU vitamin D daily | Exercise counselling provided by research centre | Exercise counselling provided by research centre |
| Bredella, et al 2021 | 1200mg elemental calcium daily  800 IU vitamin D daily | n/a | nil |
| Mitchell et al 2023 | 25OH supplementation: 400IU daily, if levels bewlow 12- 20 ng/ml 50,000 IU/ week for 2 months, for 25OH levels below 12 ng/ml 50,000 IU/ week for 3 months | n/a | n/a |
| Jarvholm et al 2023 | Multivitamin and mineral supplementation prescribed by the bariatric team | 800 calories per day for 8 weeks | Patients in the lifestyle group underwent 60mins moderate to vigorous intensity daily activity and reduced sedentary activity |
| Santos et al 2019 | 800IU Vitamin D, 250mg calcium carbonate, 125mg phosphorous, 100mg magnesium | n/a | nil |
| Kaulfers et al 2011 | Multivitamin 1000mg calcium and 800 IU of 25OH | n/a | nil |
| Weiner et al 2020 | Patient self reported if they were taking vitamin D3 tablets | nil | Liver reduction diet |
| Nadler et al 2009 | Multivitamin with iron daily | nil | nil |
| Sachdev et al 2018 | n/a | nil | nil |
| De Peppo et al 2008 | nil | nil | Calorie restriction (700- 1000 kcal) |
| Beamish et al 2017 | Multivitamin containing 1000mg calcium, 800IU vitamin D, daily mineral supplements and vitamin B12 | nil | nil |

# Supplementary file 6a: Newcastle Ottowa Score for nonrandomized studies

| **Major Components** | **Response options** | **Mitchell et al 2022** | **Nadler et al 2009** | **Sachdev et al 2018** | **Misra et al 2020** | **De Peppo et al 2008** | **Beamish et al 2016** | **Santos et al 2019** | **Bredella et al 2021** | **Kaulfers et al 2010** | **Misra et al 2020** | **Weiner et al 2020** | **Jarvholm et al 2023** |
| --- | --- | --- | --- | --- | --- | --- | --- | --- | --- | --- | --- | --- | --- |
| Selection |  |  |  |  |  |  |  |  |  |  |  |  |  |
| 1. Representativeness of the exposed cohort |  |  |  |  |  |  |  |  |  |  |  |  |  |
| 1) truly representative of the average paediatric obese patients in the community | ☆ | ☆ | ☆ |  | ☆ |  | ☆ |  |  |  | ☆ | ☆ | ☆ |
| 2) somewhat representative of the average paediatric obese patients in the community | ☆ |  |  | ☆ |  | ☆ |  | ☆ | ☆ | ☆ |  |  |  |
| 3) selected group of users eg nurses, volunteers | / |  |  |  |  |  |  |  |  |  |  |  |  |
| 4) no description of the derivation of the cohort | / |  |  |  |  |  |  |  |  |  |  |  |  |
| 2. Selection of the non exposed cohort |  |  |  |  |  |  |  |  |  |  |  |  |  |
| 1) drawn from the same community as the exposed cohort | ☆ | ☆ |  |  | ☆ |  |  |  |  |  | ☆ |  | ☆ |
| 2) drawn from a different source | / |  |  |  |  |  |  |  |  |  |  |  |  |
| 3) no description of the derivation of the non exposed cohort | / |  |  |  |  |  |  |  |  |  |  |  |  |
| 3. Ascertainment of exposure |  |  |  |  |  |  |  |  |  |  |  |  |  |
| 1) secure record (eg surgical records) | ☆ | ☆ | ☆ | ☆ | ☆ | ☆ | ☆ | ☆ | ☆ | ☆ | ☆ | ☆ | ☆ |
| 2) structured interview ¯ | ☆ |  |  |  |  |  |  |  |  |  |  |  |  |
| 3) written self report | / |  |  |  |  |  |  |  |  |  |  |  |  |
| 4) no description | / |  |  |  |  |  |  |  |  |  |  |  |  |
| 4. Demonstration that outcome of interest was not present at start of study |  |  |  |  |  |  |  |  |  |  |  |  |  |
| 1) yes | ☆ | ☆ | ☆ | ☆ | ☆ | ☆ | ☆ | ☆ | ☆ | ☆ | ☆ | ☆ | ☆ |
| 2) no | / |  |  |  |  |  |  |  |  |  |  |  |  |
| Comparability* |  |  |  |  |  |  |  |  |  |  |  |  |  |
| 5. Comparability of cohorts on the basis of the design or analysis (can be given 2 stars) |  |  |  |  |  |  |  |  |  |  |  |  |  |
| 1) study controls for baseline bony demographics (select the most important factor) | ☆ | ☆ |  |  | ☆ |  | ☆ | ☆ | ☆ | ☆ | ☆ | ☆ | ☆ |
| 2) study controls for any additional factor (This criteria could be modified to indicate specific control for a second important factor.) | ☆ |  |  |  |  |  |  |  |  |  |  |  | ☆ |
| 6. Assessment of outcome |  |  |  |  |  |  |  |  |  |  |  |  |  |
| 1) independent blind assessment | ☆ |  |  |  |  |  |  |  |  |  |  |  |  |
| 2) record linkage | ☆ | ☆ | ☆ | ☆ | ☆ | ☆ | ☆ | ☆ | ☆ | ☆ | ☆ | ☆ | ☆ |
| 3) self report | / |  |  |  |  |  |  |  |  |  |  |  |  |
| 4) no description | / |  |  |  |  |  |  |  |  |  |  |  |  |
| 7. Was follow-up long enough for outcomes to occur |  |  |  |  |  |  |  |  |  |  |  |  |  |
| 1) yes (select an adequate follow up period for outcome of interest) 1 year | ☆ | ☆ | ☆ | ☆ | ☆ |  | ☆ | ☆ | ☆ | ☆ | ☆ | ☆ |  |
| 2) no | / |  |  |  |  |  |  |  |  |  |  |  |  |
| 8. Adequacy of follow up of cohorts |  |  |  |  |  |  |  |  |  |  |  |  |  |
| 1) complete follow up - all subjects accounted for ¯ | ☆ |  |  |  | ☆ |  |  | ☆ | ☆ |  | ☆ | ☆ |  |
| 2) subjects lost to follow up unlikely to introduce bias - small number lost - > ___80_ % (select an adequate %) follow up, or description provided of those lost) | ☆ | ☆ | ☆ | ☆ |  | ☆ | ☆ |  |  |  |  |  | ☆ |
| 3) follow up rate < 80 % followed up (select an adequate %) and no description of those lost | / |  |  |  |  |  |  |  |  |  |  |  |  |
| 4) no statement | / |  |  |  |  |  |  |  |  |  |  |  |  |
| **TOTAL** |  | **8** | **6** | **6** | **8** | **5** | **7** | **7** | **7** | **6** | **8** | **7** | **8** |

# Supplementary file 6b: Modified JADDAD score for Randomized Controlled Trials

| **Study: first author and year of publication** | **Was the study described as randomised? (Y/N)** | **Method of Randomisation appropriate? (1 or -1)** | **Was the study described as blinding? (Y/N)** | **Was blinding appropriate (2, 1 or 0 points)** | **Was there a description of withdrawls and dropouts? (1 point)** | **Was there a clear description of the inclusion/exclusion criteria? Y/N)** | **Was the methods of statistical analysis described? (Y/N)** | **Was the method used to assess adverse effects described? (Y/N)** | **Overall JADDAS score,** |
| --- | --- | --- | --- | --- | --- | --- | --- | --- | --- |
| Jarvholm et al, 2023 | 1 | 1 | 1 | 1 | 1 | 1 | 1 | 1 | 8, High |

# Supplementary file 7: Funnel Plots & Eggers test for publication bias


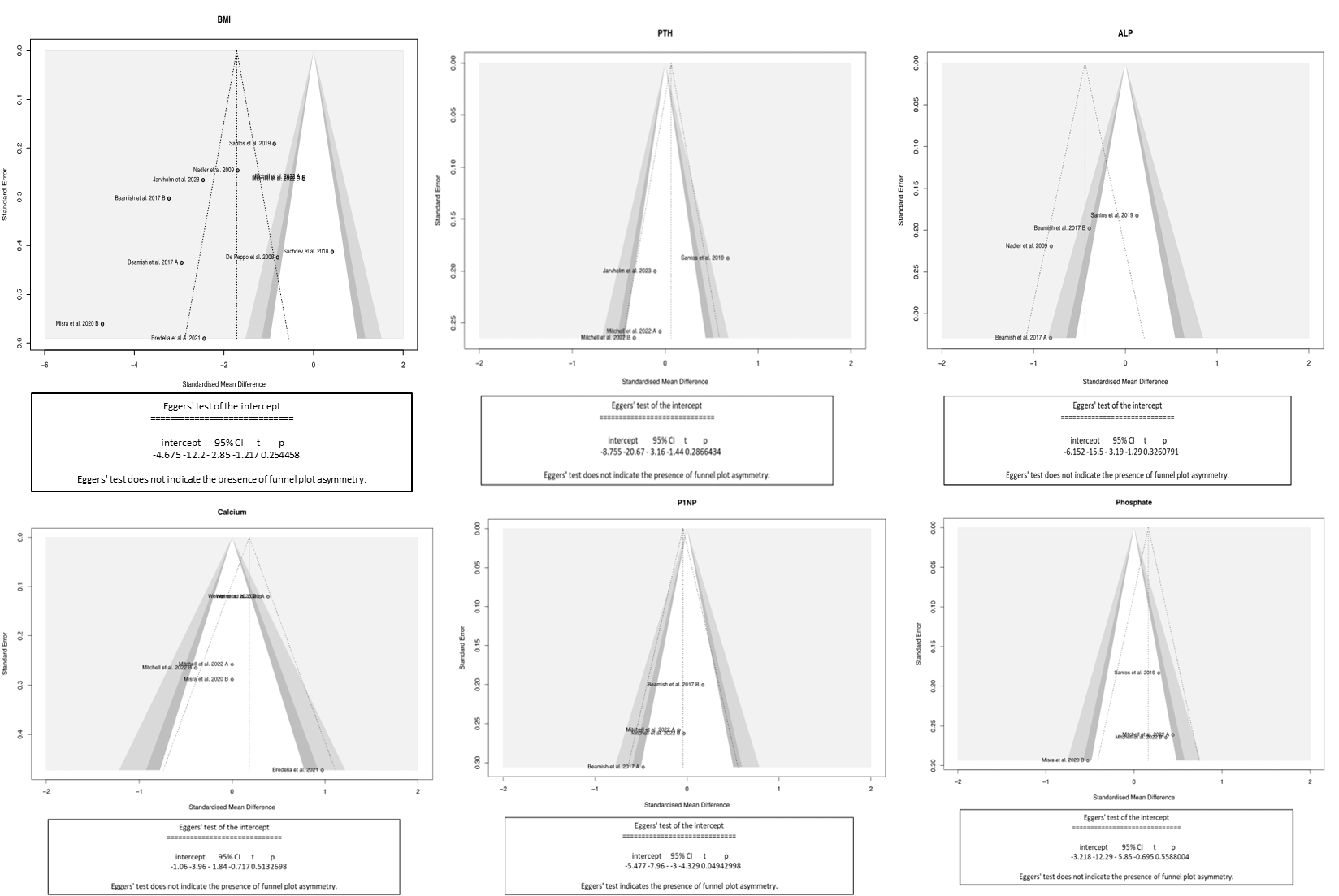


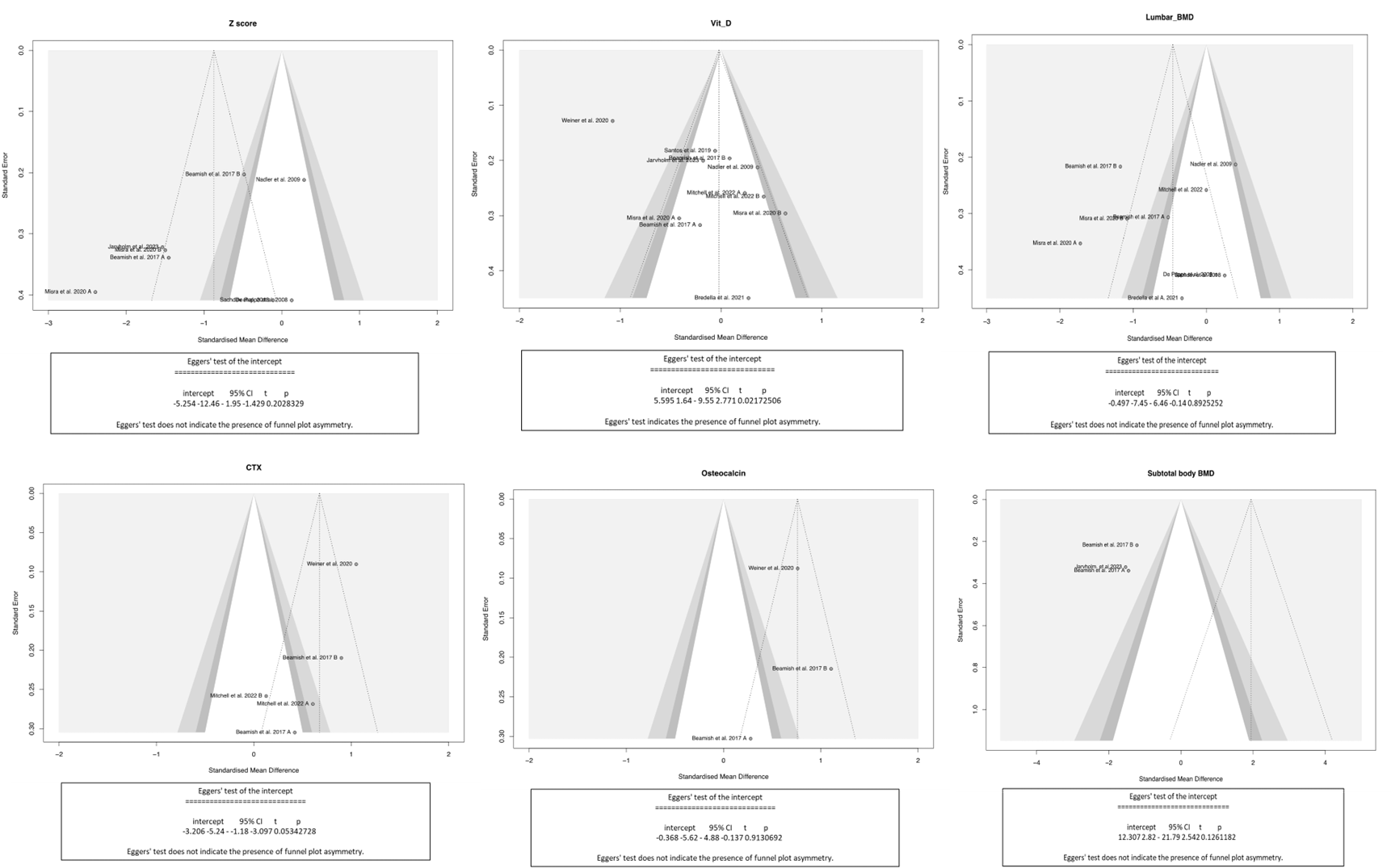

Supplement: Supplementary Material [file mmc1.docx]
